# Application of Multi-Branch Cauer Circuits in the Analysis of Electromagnetic Transducers Used in Wireless Transfer Power Systems

**DOI:** 10.3390/s20072052

**Published:** 2020-04-06

**Authors:** Milena Kurzawa, Cezary Jędryczka, Rafał M. Wojciechowski

**Affiliations:** Institute of Electrical Engineering and Electronics, Poznan University of Technology, 60-965 Poznan, Poland; milena.kurzawa@put.poznan.pl (M.K.); cezary.jedryczka@put.poznan.pl (C.J.)

**Keywords:** field-circuit model, multi-branch equivalent circuit, Cauer circuits, wireless electric power transmission

## Abstract

In this paper, the feasibility of applying a multi-branch equivalent model employing first- and second-order Cauer circuits for the analysis of electromagnetic transducers used in systems of wireless power transfer is discussed. A method of formulating an equivalent model (EqM) is presented, and an example is shown for a wireless power transfer system (WPTS) consisting of an air transformer with field concentrators. A method is proposed to synthesize the EqM of the considered transducer based on the time-harmonic field model, an optimization algorithm employing the evolution strategy (ES) and the equivalent Cauer circuits. A comparative analysis of the performance of the considered WPTS under high-frequency voltage supply calculated using the proposed EqM and a 3D field model in the time domain using the finite element method (FEM) was carried out. The selected results of the conducted analysis are presented and discussed.

## 1. Introduction

The dynamic development of electrical and electronic equipment commenced in the second half of the 20th century, and the pursuit of its continuous improvement meant that, today, one of the most developed research areas is related to the search for new technologies and methods of wireless electric energy transmission. Among the many currently offered methods of wireless energy transmission [1,2,3], the most frequently used method is the transfer of electric energy using a higher frequency electromagnetic field. The benefits of this method of energy transfer include the high reliability of the systems and user convenience associated with the “full” mobility of electrical and electronic devices. Because of the advantages discussed above, wireless power transfer systems (WPTS) have found a wide range of uses in many engineering and home applications. WPTSs are used in charging systems for electronic devices [4,5]; in robotics to the supply arms of a series of manipulators [6]; and in medicine for charging batteries of devices supporting human organs [7] or batteries of systems supplying medical sensors used in diagnostics [8,9]. These systems are also widely used in today’s dynamically developing field of electromobility [10,11].

Initially, methods using lumped parameter models [12], commonly called circuit models (CM), were primarily used to analyze the performance of wireless energy transfer systems using higher frequency electromagnetic fields. Over time, models with higher computational reliability, i.e., field models (FM) using the finite element method (FEM) [13,14], were introduced for WPTS analysis. When analyzing systems consisting of electromagnetic transducers with simple geometry, the commonly used approach is based on two-dimensional (2D) models. In the case of systems characterized by a complex magnetic circuit structure, three-dimensional (3D) models are implemented. The advantage of using 3D models in relation to 2D models is the higher reliability of the obtained results. Unfortunately, 3D models are characterized by much higher computational complexity than 2D models. Therefore, 3D models are currently used only when the accuracy of the obtained results is more crucial than the speed of calculations, i.e., usually at the design and optimization stages of very complex structures of electromagnetic transducers fed by higher frequency sources [15,16]. However, it should be mentioned here that, in parallel with field models, circuit models are still being developed. It is generally accepted that CMs are used wherever short calculation times are required and when the response of the system under consideration must be almost immediate, e.g., in the control systems of a WPTS. Circuit models are characterized by lower computational complexity than field models because of the adopted assumptions and simplifications. However, the accuracy of results obtained on the basis of the CM is often unsatisfactory, especially in the analysis of systems operating at high frequencies. In order to increase the reliability of calculations based on the lumped parameters of the considered systems, the values of their parameters can be determined using field models. Nevertheless, the values of lumped parameters are commonly determined for a predetermined value of the power source frequency and given values of currents and/or voltages. It should be noted that in a WTPS operating at a high frequency due to the presence of eddy currents and/or displacement currents, the lumped parameters of the studied systems are dependent on the frequency of the power source. Therefore, the lumped parameters of such systems should be determined for each of the considered frequency values by creating a series of independent CMs valid for a selected frequency value.

As a result of the aforementioned inconveniences concerning the use of field models and lumped parameter models, new methods allowing for the analysis of electromagnetic devices powered by higher frequency sources are still being sought. Currently, in the analysis of these systems, equivalent models (EqM) combining the advantages of both FMs and CMs are beginning to be employed. Such equivalent models can be formed by multi-branch Foster or Cauer circuits to represent the frequency-dependent characteristics of the values of the lumped parameters of the electromagnetic transducers implemented in WPTSs. It was demonstrated in [17], among other studies, that the use of these models allows for shortening the calculation time while maintaining enough reliability of the obtained results.

**Figure 1:**
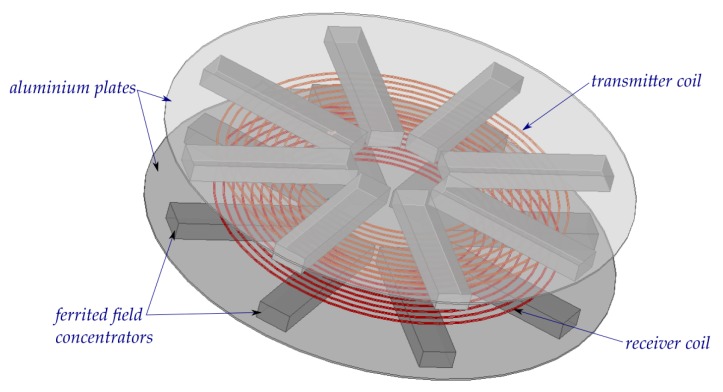
View of considered electromagnetic transducer of a wireless power transfer system (WPTS).

In this article, the authors discuss an algorithm for formulating an equivalent electromagnetic model by using an example of an air transformer consisting of field concentrators (see Figure 1), which are integral components of WPTSs. For the synthesis of the discussed EqM of the studied transducer, multi-branch Cauer circuits of the first and second order were used. Their parameters were determined using an optimization algorithm and the time-harmonic field model. Selected results of the calculations obtained for the developed EqM model for selected power source frequency values are given. The obtained results were compared with the results obtained for the time-dependent 3D field model elaborated in the professional FEM package ANSYS Maxwell.

## 2. Equivalent Model Based on Multi-Branch Cauer Circuits

**Figure 2:**
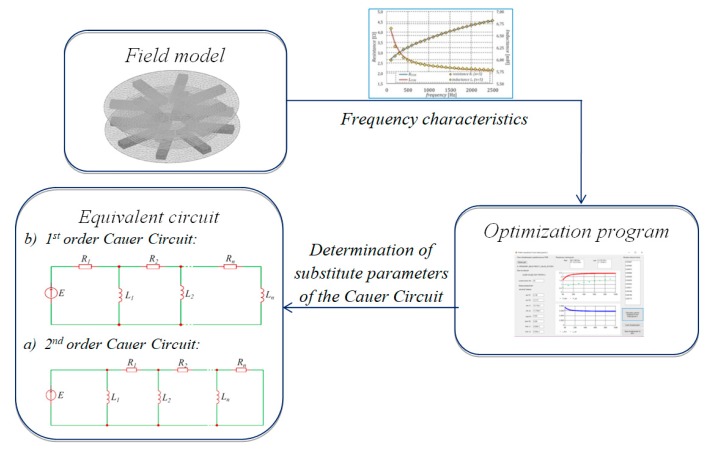
Algorithm for equivalent model (EqM) synthesis.

To synthesize an EqM of the system with an electromagnetic field, using multi-branch first- and second-order Cauer circuits, the characteristics representing the resultant impedance of the considered system as a function of the frequency of the power source must be determined as an initial step. Usually, time-harmonic field models [18] are used to determine these characteristics. The algorithm for formulating the equivalent model is given in Figure 2. Here, to calculate the EqM parameters of the considered system, a developed program employing the multi-stage approach of FEM [19] with the formulation of the combined complex potentials Ω-***T***-***T***_0_ was used. It should be mentioned that the applied approach, thanks to its universality, not only allows for the analysis of the magnetic field distribution, taking into account the distribution of eddy currents in massive conductive elements (in the considered system, for example, the influence of eddy currents in the ferrite field concentrators), but also allows for consideration of the influence of induced currents in conductive multi-connected domains (i.e., WPT system windings) on magnetic field distribution. In the proposed approach, the interpolation functions of the edge elements are employed to describe the gradient vector of the potential Ω, while the interpolation functions of the facet elements are used to describe the current vector potentials ***T*** and ***T***_0_ [19]. The equations of the Ω-***T***-***T***_0_ method lead to the formulation of a coupled system that comprises (a) a magnetic edge network (MEN) [20] and (b) an electrical facet network (EFN) [20]. A detailed description of the method of formulating FEM equations using electrical vector potentials ***T***-***T***_0_ is given in [19,21,22,23,24]. Finally, a system of matrix equations with the following compact form was obtained:

(1) $$\left[\begin{array}{ccc} k_{n}^{T}\Lambda k_{n} & k_{n}^{T}\Lambda & k_{n}^{T}\Lambda z_{0}\\ j\omega \Lambda k_{n} & R_{\rho o}+j\omega \Lambda & R_{w}^{T}\\ j\omega z_{0}^{T}\Lambda k_{n} & R_{w} & R_{DC}+j\omega z_{0}^{T}\Lambda z_{0} \end{array}\right]\left[\begin{array}{c} \Omega \\ i_{m}\\ i_{c} \end{array}\right]=\left[\begin{array}{c} 0\\ 0\\ u_{c} \end{array}\right],$$

where *j* is the unit imaginary number, ω represents the electrical angular velocity (ω = 2π*f*; *f*—frequency), **Λ** is the branch permeance matrix of the MEN, ***R***_ρo_ is the loop resistance matrix of the EFN, and ***k****_n_* is the nodal incidence matrix. Furthermore, matrix ***z***_0_ describes the winding in the space of the edge elements, ***R****_DC_* is the loop resistance matrix for the loops of an external circuit (in the analyzed example, this matrix represents the winding resistance values determined for direct current DC), and ***R****_w_* represents the mutual resistances between the loops of the EFN and external circuit loops. The vectors **Ω**, ***i****_m_* and ***i****_c_* represent nodal values of the potential Ω and the edge values of potentials ***T*** and ***T***_0_, respectively. It should be added that in the considered example, the vector ***i****_m_* describes the distribution of eddy currents induced in the coils, as well as in the massive conductive elements of the studied WPTS, while vector ***i****_c_* represents the currents induced in paths with a determined direction of current flow around the inconsistencies of the coils of the considered system. Finally, ***u****_c_* is the vector representing the values of voltages in the output terminals of the air transformer with field concentrators of the considered system.

Here, in order to determine the matrix ${\underline{Z}}^{FEM}$, which describes the impedance matrix for the studied system, the authors applied the following formula:

(2) $${\underline{Z}}^{FEM}\left(\omega \right)=R_{DC}+j\omega z_{o}^{T}\Lambda z_{0}-\left[\begin{array}{cc} j\omega z_{o}^{T}\Lambda k_{n} & R_{co} \end{array}\right]\cdot {\left[\begin{array}{cc} k_{n}^{T}\Lambda k_{n} & k_{n}^{T}\Lambda \\ j\omega \Lambda k_{n} & R_{\rho o}+j\omega \Lambda \end{array}\right]}^{-1}\cdot \left[\begin{array}{c} k_{n}^{T}\Lambda z_{0}\\ R_{co}^{T} \end{array}\right].$$

After the multiplication and addition operations of Equation (2), the matrix ${\underline{Z}}^{FEM}$ can be reduced to a simpler, more compact form:

(3) $${\underline{Z}}^{FEM}\left(\omega \right)=\left[\begin{array}{cc} R_{1}+j\omega L_{1}+\frac{R_{loss}^{-1}}{\left(R_{loss}^{-2}+{\left(\omega M\right)}^{-2}\right)} & {\left(R_{loss}^{-1}-j{\left(\omega M\right)}^{-1}\right)}^{-1}\\ {\left(R_{loss}^{-1}-j{\left(\omega M\right)}^{-1}\right)}^{-1} & R_{2}+j\omega L_{2}+\frac{R_{loss}^{-1}}{\left(R_{loss}^{-2}+{\left(\omega M\right)}^{-2}\right)} \end{array}\right],$$

where *R*_1_ and *R*_2_ represent the resistance of the WPTS windings; *L*_1_, *L*_2_ and *M* represent the self- and mutual inductance of the WPTS windings. Finally, *R_loss_* is the equivalent resistance representing the additional losses in the system as, for example, losses in massive conductive elements, as well as losses in the ferromagnetic components.

**Figure 3:**
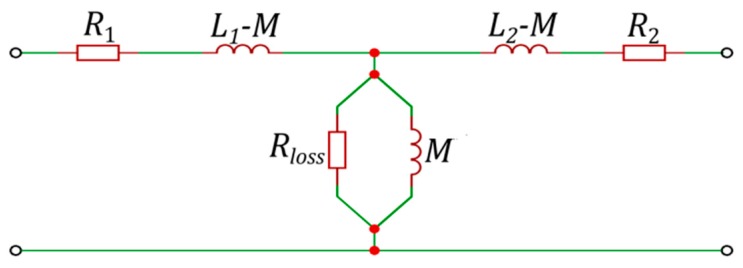
The classical equivalent circuit for the transformer of a WTPS.

The above-mentioned parameters in Equation (3) can be equated to the parameters of the classic transformer equivalent circuit (EC) (see Figure 3), which is used very often in WPTS analyses. However, it should be noted that in the majority of cases discussed in the available literature, the values of the parameters of this EC are assumed to be constant and independent of the frequency of the power source. As discussed, because of eddy current and displacement current phenomena, the values of EC parameters of the WPTS calculated on the basis of Equation (2) depend on the frequency of the power source. Therefore, a more precise description of electromagnetic phenomena in the studied systems is required. The obtained dependencies (characteristics) are used as input data in the next stage of formulating the multi-branch equivalent models.

**Figure 4:**
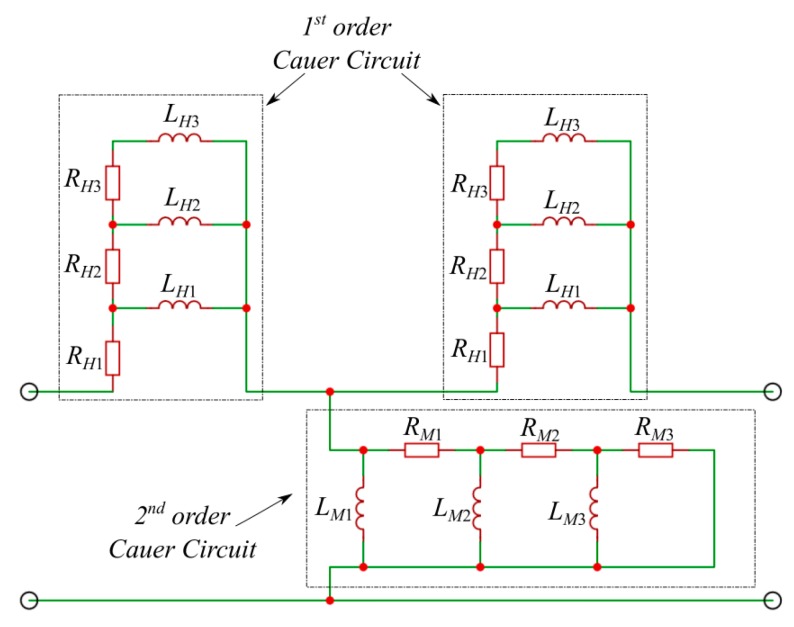
The proposed equivalent circuit for the transformer of the WPTS.

Next, on the basis of the determined frequency characteristics, the optimal values of the EqM parameters are sought. For the considered type of air transformer, the proposed equivalent circuit shown in Figure 4 was employed.

In order to determine the values of the *R_k_* and *L_k_* parameters of particular branches of the considered Cauer circuits, the minimum of the following functional should be found:

(4) $$ℑ\left(R,L\right)={\left(\sum_{i}^{N}{\left|\frac{Re\left({\underline{Z}}_{S}^{FEM}\left(\omega_{i}\right)-\underline{Z}\left(\omega_{i},R,L\right)\right)}{Re\left({\underline{Z}}_{S}^{FEM}\left(\omega_{i}\right)\right)}+\frac{Im\left({\underline{Z}}_{S}^{FEM}\left(\omega_{i}\right)-\underline{Z}\left(\omega_{i},R,L\right)\right)}{Im\left({\underline{Z}}_{S}^{FEM}\left(\omega_{i}\right)\right)}\right|}^{2}\right)}^{\frac{1}{2}},$$

where the symbol *Z* describes the total impedance representing the appropriate first- or second-order multi-branch Cauer circuit; ***R***(***R*** = [*R*_1_, *R*_2_, ... *R_n_*]) and ***L***(***L*** = [*L*_1_, *L*_2_, ..., *L_n_*]) represent single-column matrices containing the values of the desired resistances and inductances of particular branches of a given Cauer circuit, where *R_k_*, *L_k_* > 0. The symbol ${\underline{Z}}_{s}^{FEM}$ denotes the impedance of a given branch of the circuit from Figure 3 that is correspondingly related to the given branch of the circuit from Figure 4 and determined on the basis of an FE model. Depending on the branch circuit under consideration, the subscript *S* equals *H* when the horizontal branches of the equivalent circuit are considered, and it equals *M* when the impedance representing the vertical (magnetizing) branch is calculated. *N* indicates the number of samples used for the identification of the given Cauer circuit for the *i*th pulsation value of the power source ω*_i_*, while the number of branches of a given Cauer circuit is denoted by *n*.

To determine the parameters of the EqM, an optimization algorithm that combines elements of the evolutionary strategy with the operators of the genetic algorithm was used. The elaborated optimization algorithm consists of a three-level block system in which, apart from the elements combining the ES with genetic algorithm (GA) operators, the authors’ procedure was implemented, allowing for a gradual narrowing of the search area. In order to improve the convergence of the developed optimization procedure, the new operator (which can be understood as an inflow of “new blood” to the population) was introduced to the GA. The authors used their own developed software for the optimization, described in detail in [25]. The determined optimal values of EqM parameters were then implemented in the circuit model of the considered WPTS.

The values of the determined parameters for the first- and second-order Cauer circuits and the comparison of the impedance vs. frequency characteristics of the considered system are presented and discussed in Section 3.

## 3. Results

**Figure 5:**
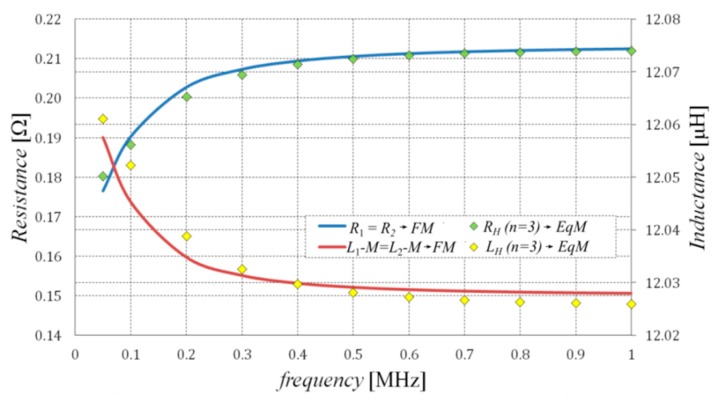
Comparison of the equivalent impedance components obtained for the horizontal branches of the transformer equivalent circuit using the first-order Cauer circuit (*n* = 3) and field model.

**Figure 6:**
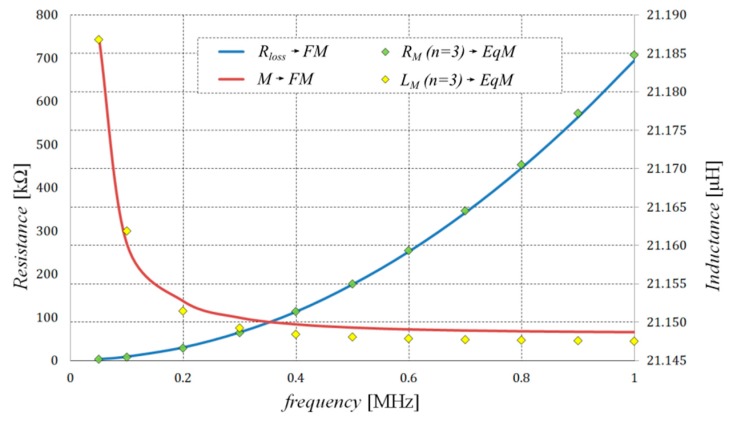
Comparison of the equivalent impedance components obtained for the magnetizing branch of the transformer equivalent circuit using the second-order Cauer circuit (*n* = 3) and field model.

The effectiveness of the proposed method of synthesis of EqM was tested through an analysis of the wireless power transmission system performance. The considered system consists of two identical coils equipped with field concentrators (see Figure 1). It was assumed that both considered coils have 10 turns and that the field concentrators are made from ferrite (PC 44) and placed on the aluminum plate. One of the coils of the considered transformer acts as a transmitter (*T*), which is supplied by the voltage source, while the second one, the receiver (*R*), is connected to the resistance load. In respect of the proposed algorithm of the formulation of an equivalent model of the WPTS described in Section 2, first, the values of lumped parameters of the WPTS were calculated in accordance with (3) using the time-harmonic field model. Then, the values of the obtained parameters were applied in order to determine the resistance and the inductance values that describe the particular branches of the considered Cauer circuits minimizing the functional given in Equation (4). Since both coils are identical in the tested system, the values of the parameters of the horizontal branches (i.e., first-order Cauer circuits) are also the same (see Figure 4). Table 1 and Table 2 summarize the values of inductance and resistance obtained by the optimization process for the first- and second-order Cauer circuits, which represent the horizontal and the magnetizing branches of the applied model, respectively. On the basis of a number of performed testing calculations, it was found that the number of branches (*n* = 3) of the employed Cauer circuits was enough to achieve negligible differences between the FM and EqM results. While conducting this research, the authors noted that increasing the number of branches *n* to more than three did not increase the accuracy of the model. The comparisons between frequency dependencies that describe the equivalent circuit parameters for the horizontal branches (obtained on the basis of the field model and the optimized EqM parameters) are shown in Figure 5, while corresponding comparisons of parameters that represent the magnetizing branch are shown in Figure 6. It should be noted that satisfactory concordance between FM and the proposed EqM results was achieved in the whole studied range of frequencies of the supply source.

Next, to test the effectiveness and reliability of the formulated EqM, the waveforms of receiver and transmitter currents, *I_R_* and *I_T_*, respectively, were determined for two different values of the frequency of the power source. The value of the amplitude of the supply voltage for both considered cases was assumed to be equal to 24 V. The system was loaded with a resistance of 120 Ω. In order to eliminate the influence of the leakage inductance of the coil in the WPTS, the additional *C*_1_ and *C*_2_ compensation capacitances were introduced. The values of these capacitances were calculated for each frequency separately on the basis of the resonance condition [26].

**Figure 7:**
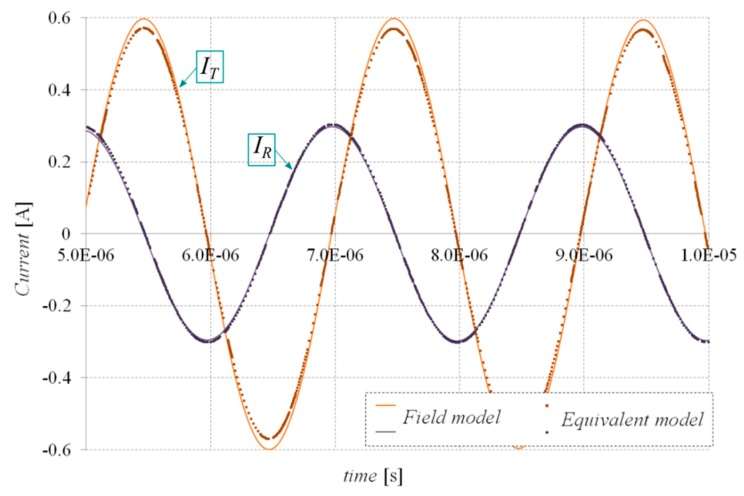
Comparison of receiver and transmitter currents of the WPTS determined by 3D FEA and the proposed equivalent model for the frequency of a supply source equal to 500 kHz.

**Figure 8:**
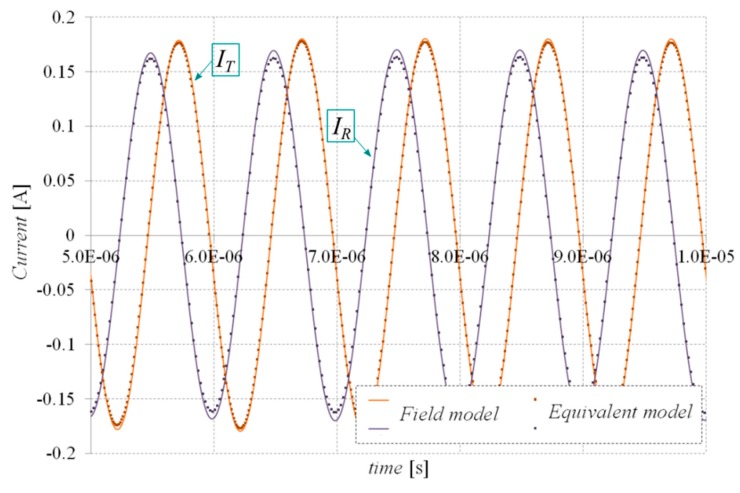
Comparison of receiver and transmitter currents of the WPTS determined by 3D FEA and the proposed equivalent model for the frequency of a supply source equal to 1 MHz.

Calculated by means of the EqM, the current waveforms *I_R_* and *I_T_* were compared with waveforms calculated by employing a detailed 3D transient field model developed in the ANSYS Maxwell environment. The comparison of the *I_R_* and *I_T_* waveforms (determined by 3D finite element analysis (FEA) and the proposed EqM) for power source frequencies of 500 kHz and 1 MHz are shown in Figure 7 and Figure 8, respectively. The time required to determine EqM parameters (including the Ω-***T***-***T***_0_ field calculations and the optimization process) was about three hours, depending on the initial values adopted in the optimization algorithm. The calculation time of the waveforms shown in Figure 4 and Figure 5 using the EqM was below 20 s, whereas obtaining these waveforms by 3D FEA took over nine hours for both cases (the benchmark calculations were performed on the same hardware, i.e., an HP Z800 Workstation). The superiority of EqM over detailed 3D FEA is especially visible when analyses need to be repeated for different input parameters, i.e., supply waveforms. This is because the amount of time required to determine the EqM parameters is spent only once at the beginning of calculations.

In the course of their work, the authors also performed a comparative analysis of the obtained current waveforms, in which the percentage difference between the results of FM and EqM were determined for each of the considered current waveforms using the formula given in [18]:

(5) $$\varepsilon_{\Delta I}=\frac{\sum_{i=1}^{n}{\left(I_{i}^{FM}\left(t_{i}\right)-I_{i}^{EqM}\left(t_{i}\right)\right)}^{2}}{\sum_{i=1}^{m}{\left(I_{i}^{FM}\left(t_{i}\right)\right)}^{2}},$$

where *m* represents the number of samples in the time domain, and $I_{i}^{FM}\left(t_{i}\right)$ and $I_{i}^{EqM}\left(t_{i}\right)$ are the current values obtained for the FM and the EqM in the time domain, respectively. The obtained results are reported in Table 3.

On the basis of the presented comparison, it can be stated that a very good concordance between the FM and the EqM results was achieved.

## 4. Conclusions

This paper proposes a method and an algorithm for formulating an equivalent model using first- and second-order Cauer circuits for an air transformer whose field concentrators are applied in wireless power transmission systems. Comparative analyses of WPTS transmitter and receiver current waveforms determined by means of the proposed equivalent model (EqM) and the “full” time-dependent 3D finite element model were carried out. The superiority of the EqM over the detailed 3D FEA in terms of the computational complexity was demonstrated. For the presented analysis of the considered transducer performance, the use of the EqM shortened the calculation time more than 1000-fold compared to calculations made using the time-dependent 3D FE model. Moreover, the conducted analysis showed practically negligible differences between the obtained results. Even taking into account the time needed to formulate an equivalent model and perform all additional calculations, high-accuracy results were obtained three times faster than those obtained by performing the full 3D FEA. Currently, the authors are focused on developing an algorithm that can reduce the time needed to synthesize an equivalent model, determining the optimal values of the parameters of Cauer circuits using the Pade via Lanczos algorithm (*PvL*) [27] or the proper orthogonal decomposition method (*POD*) [28]. Preliminary results show that the time needed to determine the EqM parameters can be reduced to an hour.

## Figures and Tables

**Table 1:** The values of resistances and inductances calculated for the Cauer circuit representing the horizontal branches of the transformer equivalent circuit.

**R_H1_ [Ω]**	**R_H2_ [Ω]**	**R_H3_ [Ω]**
0.176	1450.0	1837.0
**L_H1_ [μH]**	**L_H2_ [mH]**	**L_H3_ [mH]**
12.065	154.170	3.651

**Table 2:** The values of resistances and inductances calculated for the Cauer circuit representing the magnetizing branches of the transformer equivalent circuit.

**R_M1_ [Ω]**	**R_M2_ [Ω]**	**R_M3_ [Ω]**
1300.34	34.516	3.141·10^9^
**L_M1_ [μH]**	**L_M2_ [mH]**	**L_M3_ [mH]**
21.239	572.919	4.928

**Table 3:** Comparison of the difference ε_Δ*I*_ between results obtained for the finite model (FM) and EqM calculations.

Frequency	$\varepsilon_{\Delta I}\left(I_{R}\right)$	$\varepsilon_{\Delta I}\left(I_{T}\right)$
500 kHz	0.0469	0.0198
1 MHz	0.0217	0.0363
